# Natural killer cell functional dichotomy: a feature of chronic viral hepatitis?

**DOI:** 10.3389/fimmu.2012.00351

**Published:** 2012-11-26

**Authors:** Mario U. Mondelli, Barbara Oliviero, Dalila Mele, Stefania Mantovani, Chiara Gazzabin, Stefania Varchetta

**Affiliations:** ^1^Research Laboratories, Department of Infectious Diseases, Fondazione Istituto di Ricovero e Cura a Carattere Scientifico Policlinico San MatteoPavia, Italy; ^2^Department of Internal Medicine, University of PaviaPavia, Italy

**Keywords:** IFN-α, IFN-γ, HCV, HBV, liver diseases

## Abstract

Natural killer (NK) cells are involved in innate immune responses to viral infections either via direct cytotoxicity which destroys virus-infected cells or production of immunoregulatory cytokines which modulate adaptive immunity and directly inhibit virus replication. These functions are mediated by different NK subpopulations, with cytotoxicity being generally performed by CD56^dim^ NK cells, whereas CD56^bright^ NK cells are mainly involved in cytokine secretion. NK functional defects are usually combined so that impaired degranulation is often associated with deficient cytokine production. Innate immunity is thought to be relevant in the control of hepatitis virus infections such as hepatitis B virus (HBV) and hepatitis C virus (HCV), and recent findings reproducibly indicate that NK cells in chronic viral hepatitis are characterized by a functional dichotomy, featuring a conserved or enhanced cytotoxicity and a reduced production of interferon (IFN)-γ and tumor necrosis factor-α. In chronic HCV infection this appears to be caused by altered IFN-α signaling resulting from increased signal transducer and activator of transcription 1 (STAT1) phosphorylation, which polarizes NK cells toward cytotoxicity, and a concomitantly reduced IFN-α induced STAT4 phosphorylation yielding reduced IFN-γ mRNA levels. These previously unappreciated findings are compatible on the one hand with the inability to clear HCV and HBV from the liver and on the other they may contribute to understand why these patients are often resistant to IFN-α-based therapies.

## INTRODUCTION

Hepatitis B (HBV) and hepatitis C (HCV) viruses are the most frequent liver pathogens responsible for chronic liver disease in over 600 million people worldwide, leading to cirrhosis and liver cancer, the main indications for liver transplantation (Davis et al., 2003; Hoffmann and Thio, 2007). Although HBV infection can be prevented by an effective vaccine, no vaccine is yet available for HCV infection.

The natural history of HCV infection is highly variable, with approximately 70% of the patients developing persistent infection and about 20% progressing to cirrhosis over the ensuing decades (Thein et al., 2008). HBV virtually always persist in the immunocompetent host as occult infection despite clinical recovery (Raimondo et al., 2008) but the rate of chronic infection is dramatically high when HBV is acquired at birth or early infancy (Liaw and Chu, 2009). Treatment with interferon (IFN)-α may, albeit rarely, result in viral clearance but more often patients face life-long antiviral suppressive therapy with the potential emergence of resistance and toxicity (European Association for the Study of the Liver, 2012).

A strong innate immune response is thought to be relevant in the achievement of the control of both viral infections. A common view is emerging from recent studies showing the existence of a natural killer (NK) cell functional dichotomy during chronic HBV and HCV infections, characterized by an increased cytolytic activity coupled to reduced IFN-γ production. In this review, we shall discuss the possible causes and consequences of such defect.

## INNATE IMMUNE RESPONSES TO HCV AND HBV

In agreement with findings in many viral infections, host adaptive immune responses largely determine whether HCV and HBV are spontaneously eradicated or persist (Rehermann and Nascimbeni, 2005) although key factors in immunopathogenesis still remain elusive. While innate immunity is thought to contribute to the control of hepatotropic viruses, most of what is known derives from experimentally infected chimpanzees, as they can be studied from the onset of infection through the course of the associated disease, with the caveat that the primate model may not be entirely representative of the human setting. Contrary to HBV infection in which no appreciable changes in innate immune response genes are detected in the liver of HBV-infected chimpanzees in the first weeks of infection, HCV seems to be able to efficiently induce IFN-α/β-response genes and is sensitive to IFNs* in vitro *(Wieland and Chisari, 2005). Yet, HCV seems to ignore innate defense mechanisms, as it replicates almost immediately after penetration into target cells, suggesting that innate immunity does not significantly contribute to the early control of virus infection. On the contrary, a complete clearance of HBV-DNA has been reported during the first weeks of infection in chimpanzee, before the onset of adaptive immunity (Guidotti et al., 1999).

Both HBV and HCV have developed strategies to evade the host immune response within hepatocytes. For instance, the NS3/4A HCV serine protease can cleave adaptor proteins such as TIR-domain-containing adaptor-inducing IFN-β (TRIF) and disturb binding of retinoic acid-inducible gene 1 (RIG-I) to IFN-β promoter stimulator 1 (IPS-1) disrupting pathogen recognition receptor (PRR) signaling, which in turn results in failure to activate IFN regulatory factor 3 (IRF3) with consequent impaired activation of downstream target genes, including IFN-β (Meurs and Breiman, 2007). Moreover, the HCV core protein can inhibit the Janus kinase (JAK)-signal transducer and activator of transcription (STAT) pathway and IFN signaling (de Lucas et al., 2005), resulting in reduced expression of IFN-stimulated genes (ISG). With respect to HBV proteins, both HBx and HBe can interfere with the innate immune system, the first inhibiting RIG-I and the melanoma differentiation-associated gene 5 (MDA5) pathways inducing downregulation of mitochondrial signaling (Wei et al., 2010), and the second via blockade of the Toll-like receptor signaling proteins TRIF-related adapter molecule (TRAM) and MyD88-adapter like (Mal), leading to the inhibition of NFκB and IFN-β activation (Lang et al., 2011).

### HOST IMMUNOGENETIC POLYMORPHISMS AND INNATE IMMUNITY IN VIRAL HEPATITIS

A recently recognized important host genetic factor associated with spontaneous (Thomas et al., 2009) and treatment-induced (Ge et al., 2009) HCV clearance is *IL28B* polymorphism. The *IL28B* gene encodes for IFN-λ3 (Chen et al., 2006) and members of IFN-λ family have been implicated in the killing of tumor target cells (Kotenko et al., 2003; Sheppard et al., 2003). Interestingly, although the cellular receptors of IFN-α and IFN-λ are different (Sheppard et al., 2003; de Weerd et al., 2007), they share the intracellular JAK–STAT signal pathway, suggesting a pathogenetic role for this molecule in this clinical setting. However, unfavorable *IL28B* single-nucleotide polymorphisms (SNPs) do not seem to be associated with specific defects of innate immune responses, although in one study rs12979860 *IL28B* TT homozygosis was associated with increased expression of the NKG2A inhibitory receptor and reduced expression of TRAIL on CD56^dim^ NK cells (Golden-Mason et al., 2011) suggesting a possible role of *IL28B *in regulating innate immune responses in HCV infection. Moreover, Suppiah et al. (2011) have shown that *IL28B* polymorphism and HLA-C alleles can have an additive effect on NK cell responses, particularly in patients treated with IFN-α-based therapies, confirming the concept of a combined role of KIR/HLA-C interactions, IFN-λ, and NK cell-mediated control of HCV infection.

Two different polymorphisms of tumor necrosis factor-α (TNF-a) alleles have been associated with chronic HBV infection of which one, TNF-a238A, is allegedly linked to an increased risk in Europeans but not in Asians (Zheng et al., 2012), while another, TNF-a857T, seems to be protective in the Asian population (Shi et al., 2012b). A study analyzing the genetic polymorphisms of different NK receptors showed that the SNP rs2617160 in NKG2D was associated with susceptibility to chronic hepatitis B in a Han Chinese population, underlying the importance of NK immune response in the control of viral infections (Ma et al., 2010).

### NK CELLS IN VIRAL HEPATITIS

Natural killer cells are an important component of innate immunity, controlling viral infections either via direct cytotoxicity or production of immunoregulatory cytokines, particularly IFN-γ and TNFα, which modulate adaptive immunity and may directly inhibit virus replication (Biron, 1997). These functions are apparently mediated by different NK subpopulations, with cytotoxicity being generally performed by CD56^dim^ NK cells, the major population of peripheral blood (PB) NK cells, whereas CD56^bright^ NK cells are mainly responsible for cytokine secretion. This reportedly rigid distribution of tasks has recently been challenged as CD56^dim^ can mediate both functions, being able to produce large amount of IFN-γ during the first hours after stimulation (De Maria et al., 2011). NK functional defects are usually combined so that impaired cytotoxicity is virtually always associated with deficient cytokine production, however, the existence of different regulatory pathways allows single functional alterations of one of the two.

Several studies focused on circulating NK cells in viral hepatitis B and C, examining their phenotype and correlating those parameters to NK cell function yielding in many cases diverging data in chronic HCV infection, with some *ex vivo* studies suggesting that reduced NK cell frequencies did not affect spontaneous or cytokine-induced cytolytic effector function (Morishima et al., 2006; Golden-Mason et al., 2008; Oliviero et al., 2009; Dessouki et al., 2010) while others showed instead deficient NK cytolytic activity (Meier et al., 2005). Similar discrepancies were also observed in HBV studies, in which reduced cytotoxicity and cytokine production (Sun et al., 2012) or intact cytotoxicity and decreased plasmacytoid dendritic cell (pDC)-induced IFN-γ production by NK cells were both recently observed (Shi et al., 2012a).

The reasons for such controversial findings are not immediately apparent, although they may be due, in part, to the heterogeneity of patients, for which some investigators attempted to control by performing an extended phenotypic and functional analysis in a substantial number of unselected patients and healthy donors.

### NK CELL FUNCTIONAL DICHOTOMY

In our own comprehensive study involving a sizeable number of patients with chronic HCV infection (Oliviero et al., 2009) we have shown increased frequencies of NKG2D- and NKG2C-expressing NK cells in HCV- and HBV-infected patients, respectively, and a decrease in the frequency of NK cells expressing KIR3DL1 in chronic HCV infection, supporting the concept of a phenotype skewed toward activation in this setting. In line with phenotypic data, NK cells from HCV positive patients responded well to cytokine stimulation displaying normal or increased cytolytic activity, while HBV patients showed a variable cytolytic response. However, in both groups, there was a major functional defect characterized by deficient NK cell IFN-γ and TNF-α production, suggesting the existence of a functional dichotomy, featuring enhanced or normal cytolytic activity and reduced cytokine production. Our data are in agreement with those of other studies in chronic HCV (Ahlenstiel et al., 2010; Dessouki et al., 2010) and HBV infections (Peppa et al., 2010; Tjwa et al., 2011). The mechanisms responsible for this defective NK function have not been completely clarified yet but available evidence suggests that NK cells are polarized toward cytotoxicity in chronic HCV infection (Oliviero et al., 2009; Ahlenstiel et al., 2011; Edlich et al., 2012) whereas in HBV infection it may be influenced by viral load and necroinflammation (Oliviero et al., 2009; Zhang et al., 2011).

Because the antiviral effect produced by cytokines is more efficient than single target cell lysis, the dysfunctional cytokine secretion shown here may be an important mechanism contributing to virus persistence. The fundamental importance of IFN-γ in the control of viral infections has indeed been shown in several studies which demonstrated it to be a powerful non-cytolytic mechanism of viral clearance from infected hepatocytes (Guidotti et al., 1999; Guidotti and Chisari, 2001). In line with this, the functional NK cell defect described above for chronic hepatitis C has been interpreted as a consequence of chronic exposure to HCV-induced IFN-α leading to chronic liver inflammation via cytotoxic mechanisms but not to viral clearance because of insufficient IFN-γ production (Ahlenstiel et al., 2010). Mechanistic insights into the cause of a reduced IFN-γ secretion by NK cells in this setting comes from recent studies indicating altered IFN-α signaling resulting from increased IFN-α-stimulated STAT1 phosphorylation, which polarizes NK cells toward cytotoxicity, and a concomitantly reduced IFN-α-induced STAT4 phosphorylation yielding reduced NK cell IFN-γ mRNA levels (Miyagi et al., 2010; Edlich et al., 2012).

As outlined above, different mechanisms would be responsible for defective IFN-γ production in chronic hepatitis B, with IL10 playing a causative role in this context (Peppa et al., 2010). Interestingly, the inhibitory effect of IL10 is only transient during acute hepatitis B and is particularly evident in the early stage of infection when viral replication reaches its acme. Of note, IL10 blockade restores NK cell ability to produce IFN-γ in actively replicating HBV-infected patients. Even more interestingly, IL10 inhibits IFN-γ production but has no effect on cytotoxicity. In addition, HBV interferes with pDC–NK crosstalk reducing IFN-γ secretion by NK cells, without affecting their cytotoxic ability (Woltman et al., 2011). It is interesting to note that in chronic HBV infection both pDC and IL10 interfere with cytokine production only, while sparing cytotoxic function. Another inhibitory molecule found to be upregulated on NK cells during CHB is the T cell immunoglobulin- and mucin-domain containing molecule-3 (Tim-3), which may also impair both NK cell functions (Ju et al., 2010).

Whether the findings obtained with PB NK cells are relevant to the liver compartment where immune-mediated chronic inflammation actually takes place remains to be elucidated.

## LIVER-INFILTRATING NK CELLS IN CHRONIC HCV AND HBV INFECTION

Intrahepatic (IH) NK cells in humans have attracted the interest of many investigators. Several studies in chronic HBV and HCV infections emphasized differences between the IH and PB compartments (Bonorino et al., 2009; Oliviero et al., 2009; Ahlenstiel et al., 2010). In those studies, a larger proportion of IH NK cells express activation molecules and TNF-related apoptosis-inducing ligand (TRAIL) compared with the PB compartment and this led many to advocate it as a proof of a pathogenetic role for NK in liver necroinflammation (Dunn et al., 2007; Ahlenstiel et al., 2010). However, the vast majority of studies in humans lack functional evaluation of IH NK cells and, therefore, it is impossible to know whether phenotypic changes actually mirror alterations in IH NK cell cytolytic potential or cytokine production. Recent data from our laboratory, for which appropriate IH NK cell controls were obtained for the first time from subjects who agreed to donate a liver tissue fragment during laparoscopic cholecystectomy, showed instead that *ex vivo* isolated IH NK cells from patients with chronic HCV infection displayed reduced degranulation ability compared to controls with apparently conserved NKG2D-mediated IFN-γ production. This apparent discrepancy with data of PB NK cells should be weighed against the fact that a different protocol was used to stimulate NK for cytokine production. Indeed, because a significant enrichment of NKG2D-expressing NK cells was observed in HCV-infected patients, which suggests a role for this activating receptor in recognition of HCV-infected hepatocytes, we used ULBP-1, one of the NKG2D ligands as stimulus (Varchetta et al., 2012). Even allowing that different stimulation protocols may have influenced data on NK cytokine secretion, it is still unclear why the cytolytic defect is apparently restricted to IH NK cells. It may be that the peculiar liver environment plays an important role in this process. Indeed, selected NK cell populations can accumulate inside the liver, as it has recently been shown (Kramer et al., 2012), which can display a unique functionality. Moreover, the relatively impaired IH NK cytotoxic function detected in our study may have different explanations related to the liver compartmentalization of the virus which may have a direct inhibitory effect on NK cell function. For instance, it is known that HCV is able to inhibit NK cells by interaction between the E2 protein and CD81 (Crotta et al., 2002; Tseng and Klimpel, 2002) and that the HCV core protein induces upregulation of MHC-I on hepatocytes (Herzer et al., 2003) and the HCV peptide 35–44 stabilizes the expression of HLA-E on liver cells inhibiting NKG2A-mediated cytolysis (Nattermann et al., 2005). It has been shown that IH levels of IL10 determine an immunosuppressive environment both in mice (Lassen et al., 2010) and humans (Accapezzato et al., 2004) and, in agreement with the aforementioned, it has been reported that IH, HCV-specific IL10-producing, non-classical regulatory CD8^+^ T cells may prevent liver damage during chronic infection (Accapezzato et al., 2004; Abel et al., 2006). This, coupled to exhaustion induced by continuous receptor engagement (Tripathy et al., 2008; Bolanos and Tripathy, 2011) would eventually lead to defective cytolytic function.

The functional cytotoxic defect observed was mirrored by a unique phenotype characterized by increased expression of activating (NKp46, NKG2D) receptors in the face of reduced TRAIL and CD107a expression, compared with controls (Varchetta et al., 2012). These findings indicate dysfunctional IH NK cell cytotoxicity associated with TRAIL downregulation in chronic HCV infection, which may contribute to virus persistence. Interestingly, contrary to healthy donors, PBMC NK cells from HCV-infected patients fail to upregulate TRAIL and CD107a when exposed to culture-derived HCV (HCVcc), suggesting an accessory cell-dependent, direct effect of the virus on TRAIL-mediated cytotoxicity (Varchetta et al., 2012). The importance of the role of TRAIL in chronic HCV infection is further emphasized by evidence that TRAIL is upregulated at the gene level in patients who have successfully responded to IFN-α treatment (Stegmann et al., 2010) and by data showing upregulation of this molecule on NK cells from healthy donors following IFN-α exposure *in vitro* (Ahlenstiel et al., 2010). These previously unappreciated findings are compatible on the one hand with the inability to clear HCV from the liver and on the other with occasional resistance to IFN-α-based therapies. A pathogenetic role for TRAIL has also been suggested in chronic HBV infection, which is characterized by a typically multifaceted clinical expression. Indeed, during periodic reactivation of liver necroinflammation associated with ALT flares, TRAIL was found to be upregulated on NK cells in the blood and in the liver and responsible for hepatocyte apoptosis following induction by IFN-α (Dunn et al., 2007). However, another study showed that HBV core protein blocks TRAIL ligand DR5 expression on hepatocytes leading to the inhibition of TRAIL-induced apoptosis (Du et al., 2009), suggesting the presence of a common mechanism of TRAIL-mediated control of virus spread in the liver in both HCV and HBV infection.

However, few studies have analyzed the human IH NK cell phenotype and function during chronic hepatitis B (Bonorino et al., 2009; Ju et al., 2010) and only one (Zhang et al., 2011) included IH NK cells from healthy donors, showing that IH NK cells are activated and hypercytolytic in this setting. New studies on IH NK cell function are needed to confirm these data and to define the role of this population in CHB.

## CONCLUDING REMARKS

**FIGURE 1:**
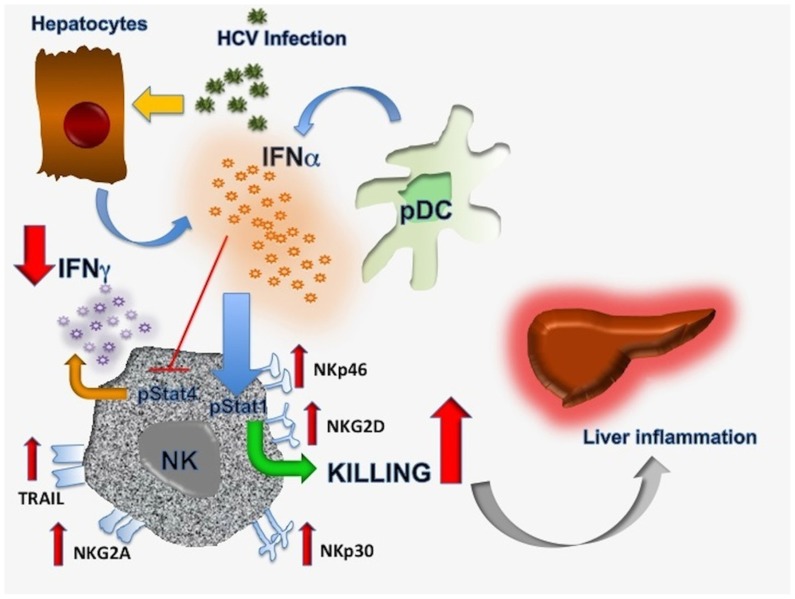
**Proposed mechanisms responsible for NK cell functional dichotomy in chronic hepatitis C infection**. Details are discussed in the text.

Available evidence indicates that impaired IFN-γ secretion is a reproducible feature of chronic hepatotropic viral infections. While mechanistic insights are by and large lacking in HBV, this defect appear to be caused by NK cell polarization toward cytotoxicity in HCV. A possible sequence of events is depicted in **Figure 1**. Hepatocytes and pDC would release substantial amounts of IFN-α as a consequence of chronic HCV infection which will preferentially stimulate STAT1 rather than STAT4 phosphorylation, resulting in reduced IFN-γ synthesis and secretion, upregulation of several NK cell activating receptors and leading to predominantly cytolytic activity. This functional dichotomy would eventually result in the inability to eliminate HCV while maintaining continuous liver inflammation. It is clear that the mechanism hypothesized here only relates to innate immunity and that cross-talk with adaptive immune responses should play a major role in this setting. Nevertheless, it does provide an attractive hypothesis for HCV persistence based on reproducible findings from several groups working in the field.

## Conflict of Interest Statement

The authors declare that the research was conducted in the absence of any commercial or financial relationships that could be construed as a potential conflict of interest.
